# Reversible and selective ion intercalation through the top surface of few-layer MoS_2_

**DOI:** 10.1038/s41467-018-07710-z

**Published:** 2018-12-11

**Authors:** Jinsong Zhang, Ankun Yang, Xi Wu, Jorik van de Groep, Peizhe Tang, Shaorui Li, Bofei Liu, Feifei Shi, Jiayu Wan, Qitong Li, Yongming Sun, Zhiyi Lu, Xueli Zheng, Guangmin Zhou, Chun-Lan Wu, Shou-Cheng Zhang, Mark L. Brongersma, Jia Li, Yi Cui

**Affiliations:** 1 Department of Materials Science and Engineering, Stanford University, Stanford, California 94305 USA; 2 State Key Laboratory of Low Dimensional Quantum Physics, Department of Physics, Tsinghua University, Beijing, 100084 P.R. China; 3 Laboratory for Computational Materials Engineering, Division of Energy and Environment, Graduate School at Shenzhen, Tsinghua University, Shenzhen, 518055 P.R. China; 4 Department of Physics, Stanford University, Stanford, California 94305 USA; 5 Stanford Institute for Materials and Energy Sciences, SLAC National Accelerator Laboratory, 2575 Sand Hill Road, Menlo Park, California 94025 USA

## Abstract

Electrochemical intercalation of ions into the van der Waals gap of two-dimensional (2D) layered materials is a promising low-temperature synthesis strategy to tune their physical and chemical properties. It is widely believed that ions prefer intercalation into the van der Waals gap through the edges of the 2D flake, which generally causes wrinkling and distortion. Here we demonstrate that the ions can also intercalate through the top surface of few-layer MoS_2_ and this type of intercalation is more reversible and stable compared to the intercalation through the edges. Density functional theory calculations show that this intercalation is enabled by the existence of natural defects in exfoliated MoS_2_ flakes. Furthermore, we reveal that sealed-edge MoS_2_ allows intercalation of small alkali metal ions (*e.g*., Li^+^ and Na^+^) and rejects large ions (e.g., K^+^). These findings imply potential applications in developing functional 2D-material-based devices with high tunability and ion selectivity.

## Abstract (web summary)

Electrochemical ion intercalation in 2D layered materials is known to occur through the material’s edges, accompanied by frequent structural deformations. Here the authors show that in MoS2 flakes where the edges have been sealed, a reversible and ion-selective intercalation occurs through the top surface via the intrinsic defects.

## Introduction

Two-dimensional (2D) materials such as graphene, hexagonal boron nitride, and transition metal dichalcogenides (TMDs) have attracted intense interest in areas of optoelectronics^1,2^, nanoelectronics^3^, and membrane separations^4,5^, due to their unique physical and chemical properties^6,7^. Molybdenum disulfide (MoS_2_) is a member of 2D layered TMDs consisting of molecular layers held together by van der Waals forces. Monolayer MoS_2_ is one of the thinnest semiconductors available and has been widely studied in electronic devices^2,3^. The maximum carrier density is on the order of 10^12^–10^14^ cm^−2^ with solid dielectric gating and ionic liquid gating^8,9^. In contrast, few-layer or bulk MoS_2_ has been mostly used in energy storage^10^ and electrocatalysis^11^. Recently, based on the same principle (intercalation/de-intercalation) as in electrochemical applications, guest species such as alkali metal ions (Li^+^, Na^+^, and K^+^) have been introduced into the large interlayer spacing (~ 0.615 nm) to manipulate and optimize the optical and electrical properties of few-layer MoS_2_^12–14^. Ion intercalation^12,15–24^ enables extremely high doping level (e.g., 6 × 10^14^ cm^−2^ in few layer graphene after Li intercalation^25^) compared with electrical gating. Such high doping levels allow new physics to be discovered, such as superconductivity^26^ and facilitates applications of few-layer MoS_2_ in optoelectronic and nanoelectronic devices. However, intercalation of ions often induces wrinkling and distortion of MoS_2_ and even irreversible structural changes^14,27,28^ that hinder its practical applications.

On the other hand, ultrathin 2D materials have been explored as novel separation membrane to realize ultrafast and high-selective sieving of gases and ions at low energy cost^4,5,29–31^. In these applications, the structural defects, interlayer spacing, or pores created by ion bombardment and oxidative etching have been used as the transport channels for the species. Manipulating ion transport through 2D material membrane via delicate electrical control, which has never been achieved before, would be much more effective and provide additional freedom for membrane designs of future functional devices.

Here, we demonstrate reversible and selective ion intercalation through the top surface of few-layer MoS_2_. We seal the edges of MoS_2_ to alleviate the structural deformation and to allow careful examination of intercalation only through the top surface. Through in situ optical and Raman measurements as well as the ab-initio density functional theory (DFT) calculations, we prove that the ions can intercalate through the intrinsic defects into the few-layer MoS_2_, and this type of intercalation is much more reversible than through the edges. The subtle electrochemical control can dramatically modify the optical and electrical properties of MoS_2_ in a reversible manner. Particularly, we obtain electron density up to 10^22^ cm^−3^ in few-nanometer-thick flakes, highest value among all the gating methods. The reversible ion intercalation and de-intercalation through top surface will benefit future material designs in highly tunable and stable 2D material-based optoelectronic and nanoelectronic devices. Furthermore, we show that the sealed MoS_2_ flakes allow intercalation of Li^+^, Na^+^ but not K^+^ and the selective intercalation through electrochemical control holds great potential in applications, such as ionic sieving and desalination of salted water.

## Results

### Electrochemical intercalation

**Fig. 1:**
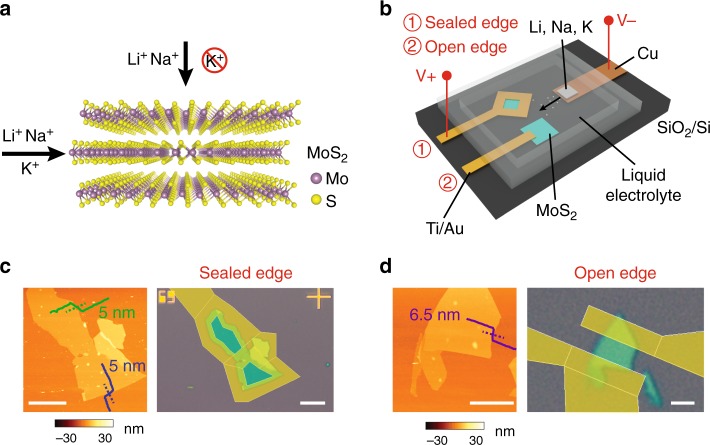
Schematic representation of the material and of the experimental setup. **a** Li^+^, Na^+^, and K^+^ intercalation into MoS_2_ through top and edge channels. **b** An electrochemical cell used to perform intercalation of Li^+^, Na^+^, and K^+^ into MoS_2_ with sealed and open edge. **c**-**d** AFM and optical microscopy images of typical MoS_2_ flakes designed for sealed edge measurement and open edge measurement, respectively. The thicknesses are typically < 10 nm, measured across three edges indicated by the dash lines. Scale bars in **c** and **d**, 5 µm

Figure 1a shows the layered structure of MoS_2_. Each layer has a plane of close-packed molybdenum (Mo) atoms sandwiched by two planes of close-packed sulfur (S) atoms (S–Mo–S). The atoms within each layer are strongly bonded by covalent interactions, whereas the interactions between layers are through weak van der Waals forces. The weak forces between the layers allow expansion of the van der Waals gap and insertion of ions. A planar battery configuration (Fig. 1b) was applied to perform electrochemical intercalation where MoS_2_ flakes and alkali metals or alkali-containing electrode materials were used as working and counter electrodes, respectively. To compare the intercalation behavior of MoS_2_ flakes with sealed edge and open edge, the Au electrodes around flake or on flake were carefully designed and patterned by electron-beam lithography (EBL) (see Methods). MoS_2_ flakes, alkali metals/salts, and electrolyte were sealed in a cell where the top transparent glass allows in situ optical observation and confocal Raman microscopy. Figure 1c, d show how sealed edge and open edge configurations are designed on two typical MoS_2_ flakes—the electrodes covering all the edges for sealed edge and that only covering part of the flakes for open edge. Atomic force microscopy (AFM) images of the flakes show that these flakes were around 6.5 nm (~10 layers) and 5 nm (~8 layers) in thickness, respectively. We also performed AFM measurements before and after the Au depositions to inspect the MoS_2_/Au morphology and interface. The clear steps of the Au metal on top of MoS_2_ edges indicated that the Au deposition was mild and uniform and successfully sealed the MoS_2_ flake without damaging the covered MoS_2_ surface (Supplementary Fig. 1).

### Li-ion intercalation into MoS_2_ through top surface and edges

**Fig. 2:**
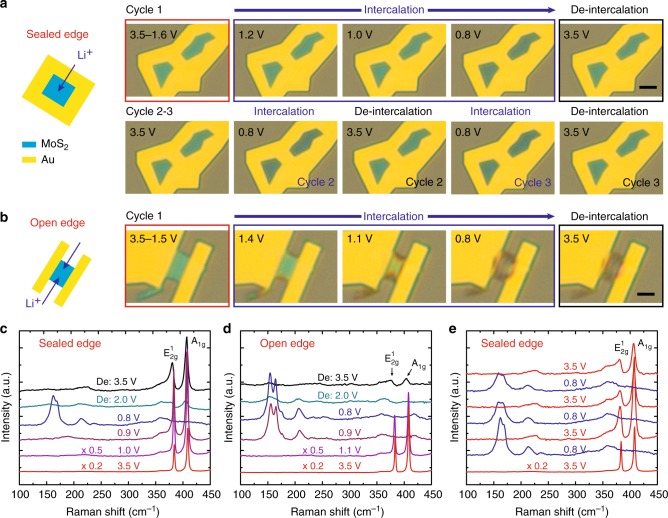
Li intercalation of MoS_2_ through the top surface and edges. **a**–**b** In situ optical microscopy images of Li intercalation into MoS_2_ through top surface and edges, respectively. Scale bars, 5 µm. **c**-**d** In situ Raman spectra of Li intercalation into MoS_2_ with sealed edge and open edge, respectively. **e** Three cycles of in situ Raman spectra of Li intercalation MoS_2_ with sealed edge. The voltages were with respect to Li/Li^+^

Figure 2 shows intercalation of Li^+^ into MoS_2_, comparing flakes with sealed edge (i.e., through top surface) and open edge (i.e., through edges). The open circuit voltage (OCV) of MoS_2_ vs. Li/Li^+^ was ~3.0 V. We gradually lowered the MoS_2_ potential with respect to Li/Li^+^ from 3.5 to 0.8 V with steps of ~0.2 V. We did not go lower than 0.8 V to avoid irreversible conversion reactions^32^. Figure 2a depicts the intercalation of Li^+^ into the sealed-edge MoS_2_ flake. When the potential of MoS_2_ was lowered to around 1.2 V, the color started to change to dark green, indicating the successful intercalation of Li^+^ into the van der Waals gap of MoS_2_ from the top surface. The flake exhibited a gradual and uniform change in color with further decrease in potential till 0.8 V, and recovered to its original bright green color when the potential was returned to 3.5 V. The intercalation and de-intercalation can be repeated for several times without disrupting the host structure (Fig. 2a, cycles 2–3). For the open-edge MoS_2_ flake (Fig. 2b), the color started to change at a relatively high potential around 1.4 V, possibly due to a lower energy barrier for the Li^+^ to initiate intercalation. The color change started from the edges toward the center, indicative of the preferential intercalation through the edges^14^. With further decrease in the MoS_2_ potential, in contrast to the sealed-edge MoS_2_, the color change exhibited non-uniform distribution with two open edges darker than the center of the flake. Furthermore, the color was not recovered when the potential was increased back to 3.5 V. The process was not reversible even when we stopped at a relatively higher potential (e.g., 1.1 V or 1.0 V) (Supplementary Fig. 2)^14^. We attribute the reversible intercalation in sealed-edge MoS_2_ to three reasons: (1) the flake was clamped and stabilized by the surrounding electrodes preventing structural deformation at the edges of the flake; (2) the diffusion pathways on the top surface are uniformly distributed and mechanically inextensible, which naturally control the intercalation homogeneity compared with the intercalation through the edges where all ions flooded into the opening van der Waals gaps; and (3) the relatively low intercalation rate of sealed-edge MoS_2_ may cause less lattice distortion and expansion. Although these factors cannot be separated, we believe the mechanical stabilization from surrounding Au electrode contributes more to the reversibility. Because the diffusion pathways on the top surface are naturally present in the MoS_2_ flakes, and these can only be triggered when all the edges are sealed.

We performed in situ Raman spectroscopy to compare the changes in MoS_2_ during Li^+^ intercalation for sealed-edge and open-edge configurations. At 3.5 V, both the Raman spectra showed two peaks located at ~384 and 408 cm^−1^, corresponding to the $E_{2g}^1$ (in-plane optical vibrations of the Mo–S bond) and *A*_1g_ (out-of-plane optical vibration of S atoms) modes of MoS_2_ (Fig. 2c, d). Besides the shift of $E_{2g}^1$ and *A*_1g_ peaks due to gating effect (Supplementary Fig. 3), the Raman spectra did not show obvious change until 1.0 V for sealed-edge MoS_2_ (Fig. 2c) and 1.1 V for open-edge MoS_2_ (Fig. 2d). Below these critical potential values, the spectra exhibited significant change and two pronounced differences can be observed by comparing the two sets of Raman spectra. First, the emergence of the new Raman modes at 154, 164, and 207 cm^−1^, often associated with the 2 H to 1 T phase transition^11,14,33^, occurs at higher potentials (~0.9 V) for open-edge MoS_2_ than for sealed-edge MoS_2_ (~0.8 V). Second, the $E_{2g}^1$ and *A*_1g_ Raman modes were largely restored and well-defined for the sealed-edge MoS_2_ after de-intercalation; in contrast, the same two Raman peaks of the open-edge nearly disappear after de-intercalation. These observations were consistent with the differences shown in the optical images and confirmed that sealed-edge MoS_2_ can show stable and reversible intercalation. By carefully comparing the peak positions of the Raman modes of 1 T phase, we found that the degree of Li intercalation in open-edge MoS_2_ was slightly higher than that of the sealed-edge MoS_2_. This is due to the lower energy barrier for intercalation in open-edge MoS_2_, which makes it hard to control the intercalation process and partially accounts for the irreversibility. Still, even for the sealed-edge MoS_2_, the modes dropped in intensity (to ~20% of the pristine MoS_2_) and showed a broadened line-width, which was likely due to the presence of residual strains^34^.

To further demonstrate the robustness of the sealed-edge MoS_2_ geometry, in situ Raman spectra corresponding to the three cycles of intercalation and de-intercalation were recorded (Fig. 2e). These spectra were highly reproducible showing well-defined $E_{2g}^1$ and *A*_1g_ Raman modes before intercalation and after de-intercalation, as well as the new Raman modes around 170 and 220 cm^−1^ after intercalation. In addition, we found both the $E_{2g}^1$ and *A*_1g_ Raman modes can be clearly identified for up to the 20 cycles of Li intercalation and de-intercalation processes (Supplementary Fig. 4), indicating the crystalline stability of sealed-edge MoS_2_. Furthermore, control experiments with MoS_2_ flake covered completely by the Au electrodes showed no signature of ion intercalation (Supplementary Fig. 5); this verifies that the Au electrodes can effectively seal the edges of MoS_2_ and the ions indeed go through the top surface in sealed MoS_2_. Previous research also showed the metal electrodes could block the entry of Li^+^ from the edges^14^. Finally, to examine the uniformity of the intercalation from the top surface, we performed a series of Raman measurements on sealed-edge MoS_2_ flakes (1) on SiO_2_/Si substrate with the Si Raman peak as reference and (2) on quartz substrate from the backside accessing the bottom surface (Supplementary Fig. 6). We found that the representative Raman peaks of Si substrate were explicitly observed for all the intercalation voltages, which indicates that the excitation laser light has totally penetrated the MoS_2_ flake and reached Si substrate (Supplementary Fig. 6a). For the intercalated state, the $E_{2g}^1$ and *A*_1g_ peaks of pristine MoS_2_ were completely undetectable, while the intensity of Si peak remained unchanged. Thus, we can confirm that the sealed-edge MoS_2_ flake was uniformly intercalated through the top surface. We also fabricated the sealed-edge MoS_2_ device on a transparent quartz substrate and carried out the Raman measurements by illuminating the excitation laser light through the bottom of the substrate directly onto the sealed-edge MoS_2_ flake. When the sample was intercalated, no intrinsic $E_{2g}^1$ and *A*_1g_ peaks of pristine MoS_2_ could be detected, further confirming the uniformity of the intercalation of MoS_2_ flake from top surface (Supplementary Fig. 6b).

### Selective ion intercalation through the top surface

**Fig. 3:**
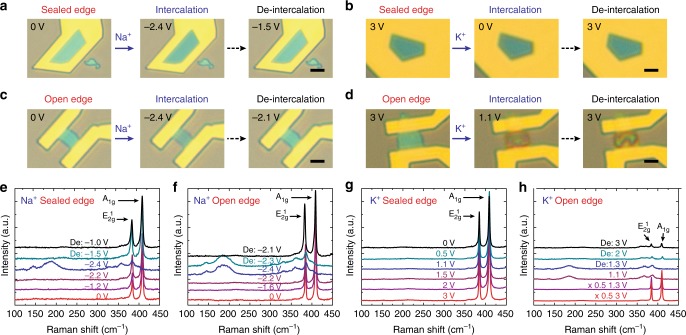
Selective intercalation from the top surface of sealed-edge MoS_2_. **a** In situ optical microscopy images show uniform color change of MoS_2_ upon Na^+^ intercalation and de-intercalation for sealed-edge MoS_2_ through the top surface. **b** The color of MoS_2_ remains unchanged when lowering the potential from 3 V to 0 V, indicating that K^+^ cannot intercalate through the top surface of sealed-edge MoS_2_. **c-d** In situ optical microscopy images show prominent color changes due to Na^+^ and K^+^ intercalation through the edges of MoS_2_. Scale bars in **a-d**, 5 µm. **e-f** In situ Raman spectra demonstrate Na^+^ intercalates into both sealed-edge MoS_2_ and open-edge MoS_2_. **g-h** In situ Raman spectra demonstrate K^+^ does not intercalate into sealed-edge MoS_2_ but intercalate into open-edge MoS_2_. The voltages in **a**, **c**, **e**, and **f** were with respect to NaCoO_2_. The voltages in **b**, **d**, **g**, **h** were with respect K/K^+^

To study the ion-selectivity of intercalation through the top surface of the MoS_2_, we tested intercalation of other alkali ions including Na^+^ and K^+^ into MoS_2_ (Fig. 3). The optical images showed very uniform color change upon Na^+^ intercalation and de-intercalation for sealed-edge MoS_2_ (Fig. 3a). In this study, we used NaCoO_2_ as the counter electrode to provide Na^+^ source and the OCV of MoS_2_ vs. NaCoO_2_ is ~0 V (the potential of NaCoO_2_ with respect to Na/Na^+^ is ~3.2 V). When the potential of MoS_2_ was lowered to ~−2.4 V, the $E_{2g}^1$ and *A*_1g_ Raman modes shifted and diminished while the new modes ~150 and 200 cm^−1^ emerged (Fig. 3e). Intercalation of Na^+^ at ~−2.4 V vs. NaCoO_2_ here (corresponding to ~0.8 V vs. Na/Na^+^) is believed to fall on the second discharge plateau^28^. When the potential of MoS_2_ was increased back to ~−1.5 V vs. NaCoO_2_, the Raman spectrum changed back to that of pristine MoS_2_. In contrast to the intercalation of Li^+^, the changes in both color and Raman spectra were also observed to be reversible for the open-edge configuration (Fig. 3c, f). The consistent observation of reversible changes in the color and Raman spectra (analogous to Li^+^ intercalation (Fig. 2)) demonstrated successful intercalation of the Na^+^ ion from the top surface into the sealed-edge MoS_2_. The relatively uniform intercalation of Na^+^ into open-edge MoS_2_ was surprising because Na^+^ (~1.16 Å) is larger than Li^+^ (~0.9 Å) in size. Based on the comparison of Raman spectra (Fig. 2d and Fig. 3f), this is probably because unlike Li^+^, the intercalation of Na^+^ did not induce a large structural deformation. In addition, we note that among all alkali metals, Na has relative weak chemical binding to various substrates including MoS_2_^35^.

On the other hand, no noticeable change was observed in the optical images upon the attempt to intercalate K^+^ into sealed MoS_2_ (Fig. 3b). The Raman spectra with different potential of MoS_2_ vs. K metal from OCV (~3.0 V) to as low as 0 V maintained the same except the very slight peak shift due to the gating effect as in the Li^+^ case, indicating no K^+^ intercalation into the sealed MoS_2_ (Fig. 3g). We suspect that this is due to the large size of K^+^ (~1.52 Å) compared with Na^+^ and Li^+^. In contrast to the sealed-edge configuration, the intercalation of K^+^ into open-edge MoS_2_ occurred when we lowered the potential of MoS_2_ vs. K metal from OCV (~3.0 V) to ~1.1 V (Fig. 3d). This is as expected because the large interlayer spacing (~0.615 nm) of MoS_2_ can accommodate K^+^ ions (~1.52 Å) when they intercalate from the edges. Still, for this open-edge MoS_2_, the color showed a non-uniform change and only partially recovered to its original state, as in the case of Li^+^ intercalation. In situ Raman spectra showed shift and diminish of the $E_{2g}^1$ and *A*_1g_ Raman modes and appearance of the new Raman modes ~140 and 190 cm^−1^ after intercalation (Fig. 3h), which was consistent with the observations in the optical images. After de-intercalation, the $E_{2g}^1$ and *A*_1g_ Raman modes shifted back with a drastically reduced intensity compared with that of the pristine MoS_2_. In addition, we show that K^+^ intercalated from the open edges and then extended into sealed areas of the same flake, which provide further evidence that K^+^ does not intercalate from the top surface of MoS_2_ (Supplementary Fig. 7).

### Analysis of intercalation pathways through the top surface

**Fig. 4:**
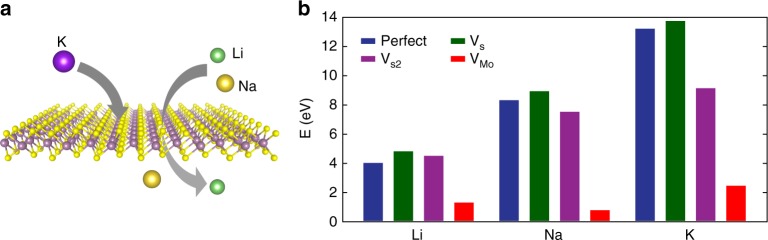
DFT calculations for alkali ions penetration through MoS_2_. **a** Schematic representation of alkali–ion intercalation through a single MoS_2_ layer. The intercalation pathways allow the penetration of Li^+^ and Na^+^ while block K^+^. **b** Energy barriers for penetration through perfect MoS_2_ (navy blue) and MoS_2_ with V_S_ (dark green), V_S2_, (purple) and V_Mo_ (red) for Li^+^, Na^+^, and K^+^, respectively

Our results have demonstrated that Li^+^ and Na^+^, but not K^+^, can be successfully intercalated into sealed-edge MoS_2_ through the top surface. To uncover the underlying mechanism, we propose that Li^+^ and Na^+^ intercalate through the natural defects^36,37^ into sealed-edge MoS_2_. We test our hypothesis by comparing the energy barriers for alkali ions to penetrate a monolayer MoS_2_ with and without defects using density functional theory (DFT) calculations (Fig. 4a). In experiment, an initial applied potential drives alkali ions to accumulate on the top of MoS_2_ surface, and these alkali ions maintain their ionic state during the intercalation and de-intercalation. In addition, due to the inversion symmetry of monolayer MoS_2_, the kinetics dominate over the thermodynamics in the process of intercalation. Therefore, the nudged elastic band method that is widely used to study the kinetic effect in electrochemical reaction^38^ is employed here. In previous research, several types of intrinsic point defects have been studied in monolayer MoS_2_ both theoretically and experimentally, including the single S vacancy (V_S_), the double S vacancy (V_S2_), single Mo vacancy (V_Mo_), and etc^39^. We first calculated the formation energy for all types of defects and found that V_S_, V_S2_, and V_Mo_ have relatively low formation energies (Supplementary Fig. 8), which are consistent with previous results^39^. Therefore, we only considered these three types of intrinsic defects and calculated the energy barriers and diffusion pathway for the intercalation of alkaline ions (Li^+^, Na^+^, K^+^) through these intrinsic defects systematically. Intercalation of alkaline ions through the perfect MoS_2_ was also considered for comparison. Figure 4b summarizes the energy barriers for Li^+^, Na^+^, and K^+^ to penetrate through perfect MoS_2_ and MoS_2_ with V_S_, V_S2_, and V_Mo_ vacancies, respectively. In the perfect MoS_2_ monolayer, the energy barriers were 4.03 eV, 8.32 eV, and 13.22 eV for intercalation of Li^+^, Na^+^, and K^+^ through the top surface, respectively. In the presence of V_S_ and V_S2_ vacancies, these values did not change significantly. In contrast, the energy barriers were significantly reduced when Li^+^, Na^+^, and K^+^ penetrate through the MoS_2_ monolayer with Mo vacancy, which were 1.30 eV, 0.79 eV, and 2.46 eV, respectively. These energy barriers for Li^+^ and Na^+^ to penetrate through MoS_2_ is comparable with the potential change (difference between OCV and intercalation voltage) in our experiments (1.30 eV vs. ~ 2.2 V and 0.79 eV vs. ~ 2.4 V). In contrast, the energy barrier for K^+^ to go through V_Mo_ is much larger (2.46 eV) compared with Li^+^ and Na^+^, explaining the unsuccessful intercalation of K^+^ even with ~3.0 V potential change in experiment. Therefore, we believe that the Mo vacancy plays an important role when the alkaline ions intercalate into MoS_2_ through the top surface (see Supplementary Figs. 9–15 for details).

We checked our MoS_2_ flakes using high-angle annular dark-field scanning tunneling electron microscopy (HAADF-STEM) and were able to observe strongly reduced brightness at certain Mo sites, which probably indicate the presence of Mo vacancies (Supplementary Fig. 16). Previous scanning tunneling microscopy study has also reported Mo-like vacancies in the MoS_2_ flakes^36^. Moreover, comprehensive investigations on the defects of monolayer MoS_2_ through high-resolution STEM have elucidated that the density of V_Mo_ is ~0.004 nm^−2^(ref ^40^). In contrast, the dominant sulfur vacancies and disulfur vacancies were reported to have higher densities (density of V_s_ ~0.12 nm^−2^ and V_S2_ ~0.017 nm^−2^)^40^. Besides the vacancy defects mentioned above, vacancy complex of Mo and three close-by sulfur atoms or disulfur pairs (V_MoS3_ or V_MoS6_) were occasionally observed in MoS_2_, but the density of V_MoS6_ was much lower than that of V_Mo_, and V_MoS3_ was too low to be counted^40^. We include both the energy barrier for intercalation and the density of the vacancies in our analysis. Considering the energy barriers for the intercalation of Li^+^, Na^+^, and K^+^ through V_S_ (V_S2_) are, respectively, 4.03 eV (4.51 eV), 8.32 eV (7.53 eV), and 13.22 eV (9.14 eV) and those through V_Mo_ are, respectively, 1.30 eV, 0.79 eV, and 2.46 eV, the relatively lower energy barriers of V_Mo_ are the determinant factor to induce the intercalation through top surface, although the density of V_Mo_ is lower than V_S_ and V_S2_. In the case of V_MoS6_, however, the extremely low density may dominate and make the intercalation unlikely, although the calculated energy barriers for the intercalation of Li^+^, Na^+^, and K^+^ through V_MoS6_ are only 0.62 eV, 0.65 eV, and 1.24 eV, respectively (Supplementary Fig. 17). This argument is supported by our experimental observation that K ions are always rejected by the surface intercalation pathways. Therefore, we believe the most preferential intercalation pathway through top surface is from the V_Mo_. Furthermore, we would like to emphaize that the real situation in the experiments is more complicated. First, the V_S_, V_S2_, and V_Mo_ may co-exsit and the S vacancies can lead to formation of Mo vacancies; second, the vacancies can evolve during intercalation due to the insertion of the ions. Nevertheless, the DFT calculations confirm that it is feasible to intercalate through the top surface into the few-layer MoS_2_ and the intercalation has selectivity.

### Reversible control of optical and electrical properties

**Fig. 5:**
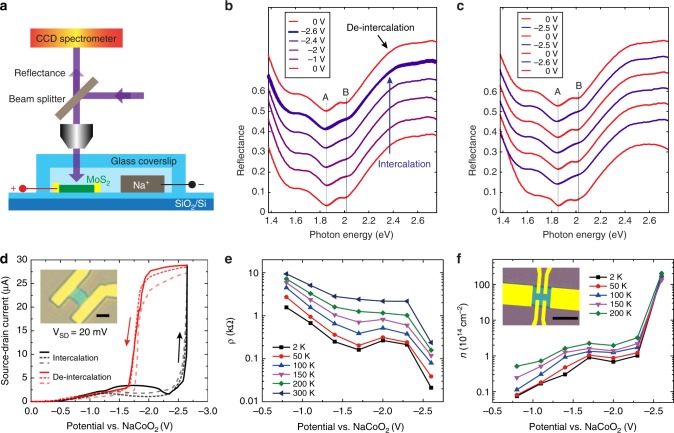
Reversible control of optical and electrical properties of MoS_2_ via Na^+^ intercalation. **a** Schematic of the optical measurement. **b** Gradual reflectance change of MoS_2_ on SiO_2_/Si via Na^+^ intercalation (spectra offset for visibility). From bottom red line to middle blue line: Na^+^ intercalation; top red line: Na^+^ de-intercalation. **c** Three cycles of reflectance change of MoS_2_ on SiO_2_/Si via Na^+^ intercalation. **d** The potential dependence of source-drain current within three intercalation/de-intercalation cycles. The bias between source and drain was V_SD_ = 20 mV at room temperature. The potential of MoS_2_ was swept from 0 V to −2.6 V and back to 0 V (vs. NaCoO_2_ counter electrode) for three cycles at a constant rate of 2 mV s^−1^. The black and red arrows indicate the sweeping directions. The inset shows the device. Scale bar, 5 µm. **e** The resistivity of MoS_2_ flake (5.5 nm) with standard Hall-bar geometry. The potential of MoS_2_ was applied at 300 K. The device was then continuously cooled down to 2 K with fixed voltages. **f** The corresponding two-dimensional electron densities at different potentials and temperatures. The inset shows the device used in (**e**) and (**f**). Scale bar, 5 µm

Both the in situ optical microscopy and Raman spectroscopy results show the high reversibility and stability of the intercalation from the top surface of the few-layer MoS_2_. Finally, we demonstrate the reversible control of both optical and electrical properties of few-layer MoS_2_ as a first step toward applications in optoelectronics and nanoelectronics devices (Fig. 5). Since Li^+^ intercalation of 2D MoS_2_ has been well-studied^13,14^, we focused on Na^+^ intercalation here. We first measured the reflectance spectra of the MoS_2_ flakes upon Na^+^ intercalation, which can also quantify the subtle color change in the optical images (Fig. 5a). For the pristine flake, two reflectance dips were observed at ~1.86 eV and 2.02 eV (red line at bottom, Fig. 5b), as a result of enhanced absorption in the MoS_2_ by the A and B excitons. These well-characterized excitons correspond to the prominent transitions between the maxima of split valence bands and the minimum of the conduction band, located at the K point of the Brillouin zone^41,42^. As we gradually lowered the potential of MoS_2_ vs. NaCoO_2_ to −2.6 V, excitonic transition B exhibited a minor blue-shift and a clear damping in intensity, due to the decrease in exciton binding energy resulted from the doped-free electrons^43,44^. The change in excitonic transition A also showed indication of damping upon ion intercalation, while this was more difficult to identify because it overlaped with a broad background Fabry–Pérot resonance in the MoS_2_–SiO_2_–Si layer stack (Supplementary Fig. 18). We performed analytical transfer-matrix calculations of the experimental geometry to resolve the Fabry–Pérot resonance besides both excitonic transitions (Supplementary Fig. 18). When we increased the potential of MoS_2_, the spectral shifts and intensity change are both fully reversed. To the best of our knowledge, this is the first report that optical properties of MoS_2_ were electrically manipulated via Na^+^ intercalation, which shows reversible and reproducible tuning over multiple cycles (Fig. 5c). Identical observations were found in the case of Li^+^ intercalation (Supplementary Fig. 19).

Next, we performed in situ electric transport measurements to show the highly tunable electrical properties via ion intercalation. We note that it is challenging to find an insulating material with high malleability, electrochemical stability, and high affinity to seal MoS_2_ edges for the electrical measurements at this point (Supplementary Fig. 20). Since Na^+^ intercalation through top surface and edges were equally reversible (Fig. 3), we employed open-edge configuration to simplify the device geometry. For the two-contact device shown in Fig. 3c, the drain-to-source current increased dramatically when the potential of MoS_2_ was continuously swept from 0 V to −2.6 V with respective to NaCoO_2_ counter electrode (Fig. 5d), because of the intercalation of Na^+^ ions into MoS_2_. When the potential was returned to around −2.0 V, the current dropped rapidly due to the extraction of Na^+^ ions from MoS_2_. Besides the current hysteresis between −1.6 V to −2.6 V arising from the over-potential effect, the current curves nearly overlapped with each other in the non-intercalated state (from 0 V to −1.6 V), which implied the restored semiconducting phase of de-intercalated MoS_2_. Moreover, the current consistency of three cycles demonstrated the stability of electrical properties after Na^+^ ion intercalation. To eliminate the effect of contact resistance and study the intrinsic transport properties, we fabricated another device with standard Hall-bar geometry and changed the applied potentials only at 300 K, higher than the ineffective temperature of electrolyte (Supplementary Fig. 21). The four-probe resistivity (Fig. 5e) reduced exponentially till nearly saturated due to the surface charging from electric-double-layer effect when the potential of MoS_2_ was continuously swept from −0.8 V to −2.3 V. As the sample was cooled down, all the resistivity decreased and displayed metallic behavior down to 2 K. When the Na^+^ ions were intercalated at the potential of −2.6 V, the resistivity decreased by an order of magnitude compared with that prior to the intercalation at −2.3 V. From the Hall effect measurements (Supplementary Fig. 22), we found that the electron density at −2.3 V can reach 1×10^14^ cm^−2^ at 2 K (Fig. 5f), consistent with that in ionic-liquid gated MoS_2_^9,45^. After Na^+^ intercalation, the electron density was increased up to 1.7×10^16^ cm^−2^, which was two orders higher than those reported using dielectric gating or liquid gating^8,9^. In 3D, it corresponds to 3×10^22^ cm^−3^ and Na_1.6_MoS_2_ in molecular formula by assuming one Na atom contributed one electron to the rigid MoS_2_ host. To the best of our knowledge, this is the highest charge-carrier density ever achieved in doped MoS_2_. Here, we use Na^+^ intercalation in open-edge MoS_2_ to show highly tunable and reversible electrical properties through ion intercalation, and the results can be extrapolated to Li^+^ or Na^+^ intercalation in sealed-edge configurations which should show even more stable performances. The reversible electrochemical control of the optical and electric performances of few layer MoS_2_ opens a new route to design highly tunable and stable 2D material-based optoelectronic and nanoelectronic devices.

## Discussion

In summary, we demonstrate that Li^+^ and Na^+^ ions can intercalate into few-layer MoS_2_ through the top surface with strongly improved control, reversibility, and stability compared with intercalation through edges. We note intercalation through top surface also applies to other similar 2D layered material systems such as MoSe_2_ (Supplementary Fig. 23). This finding is significant because electrochemical control is a powerful approach to manipulate the properties of low-dimensional materials; stable intercalation and reversible cycling are essential for accurate in situ interrogation of the physical and chemical changes during intercalation. In future, sealing the edges of 2D materials with dielectrics will allow the design of complex and tunable nanoelectronic devices with high performance and high stability. In addition, voltage-controlled selective intercalation through the top surface of the 2D materials holds great potential in developing novel ionic sieving devices that have distinct open/close (on/off) controllability via a gate voltage applied on the 2D material, besides the size and charge selectivity from common nanofiltration and desalination membranes.

## Methods

### Device fabrication

Thin MoS_2_ flakes (<10 nm) were exfoliated using the Scotch tape method onto 300-nm-thick SiO_2_/Si substrate, and electrodes (Ti/Au, 3/50 nm) were designed and patterned on flakes by electron-beam lithography and deposited by e-beam evaporation. A marker array was used for precise alignment of the electrodes to the selected flakes. The MoS_2_ sample was then transferred to an Ar-filled glovebox for cell assembly. In the case of Li^+^ and K^+^ intercalation, Li/K metal was cold pressed onto Cu foil as the counter electrode; in the case of Na^+^ intercalation, NaCoO_2_ was deposited onto an Al foil as the counter electrode. These electrodes were then sealed between a cover glass and the SiO_2_/Si substrate with evaporated Ti/Au electrode, using hot melt sealing film (Meltonix 1170–60, Solaronix), leaving two little openings for liquid electrolyte filling. There is a ~50 μm gap between the glass and the SiO_2_/Si substrate, which is then occupied by the corresponding electrolytes. The electrolytes were 1 M LiPF_6_, NaPF_6_, KPF_6_ in EC/DEC for Li^+^, Na^+^, and K^+^ intercalation, respectively. After filling the electrolyte by capillary effect, the two openings were sealed by using epoxy.

### Electrochemical intercalation

Electrochemical intercalation was performed with a Keithley 2400 sourcemeter. Constant voltage charge and discharge were used to intercalate and de-intercalate MoS_2_ flakes for in situ optical and Raman measurements.

### Transport measurements

The room temperature current between drain and source was recorded by using the Keithley 2400 sourcemeter, while another Keithley 2400 sourcemeter was used to apply charge and discharge voltage between the source and counter electrode. The low temperature transport measurements were carried out in Quantum Design PPMS-7 instrument, Janis 9 T magnet He-cryostats (base temperature 2 K), using low-frequency (5–20 Hz) AC technique by digital lock-in amplifiers (Stanford Research Systems SR830) with current-driven configuration. The charge-carrier densities were derived from Hall effect measurements.

### Raman spectroscopy

The MoS_2_ flakes were characterized using HORIBA Scientific LabRAM HR Evoluation spectrometer, with 532 nm excitation and 1800 l/mm grating. The background signals from electrolyte, cover glass, and substrates were subtracted.

### Optical measurement and simulations

Optical reflection spectra were measured using a Nikon C1 confocal microscope. Unpolarized broadband excitation from a halogen lamp and a 50 × long working distance objective (NA = 0.55) were used to illuminate the sample through the cover slip. A 30-μm pinhole was used to spatially select the reflection off the flakes, and a Princeton Instruments SpectraPro 2300i (150 l/mm, blazed at 500 nm) and PIXIS CCD camera were used to measure the spectrum. A protected silver mirror (Thorlabs) was used as a reference to correct for the system response.

### DFT calculations

The spin-polarized density functional theory (DFT) calculations were implemented in the Vienna Ab-initio Simulation Package (VASP)^46,47^. The projected augmented wave^48,49^ and Perdew–Burke–Ernzerhof (PBE) functional^50,51^ were used to describe the electron–ion interaction and exchange-correlation energy, respectively. The cutoff energy was set to 500 eV. Because of the layered structures of MoS_2_, the empirical correction method proposed by Grimme (DFT-D3) was used to simulate the van der Waals interaction^52,53^. In order to understand the intercalation process, a supercell consisting of 5×5 repeating unit cells of MoS_2_ monolayer was used, and a vacuum layer was larger than 15 Å to eliminate the spurious interaction between adjacent MoS_2_ layers. 3 × 3 × 1 Γ-centered k-points was used to sample the Brillouin zones of supercell. For each model, the structure was allowed to fully relax until the energy converged to 10^−5^eV and the residual force converged to 0.01 eV/Å per atom. The climbing image nudged elastic band (CI-NEB) method^54^ was applied to search for the transition state and determine the migration barrier of alkaline ions through the MoS_2_ monolayer.

## Electronic supplementary material

Supplementary Information

Peer Review File

## Data Availability

The data that support the findings of this study are available from the corresponding author upon reasonable request.
