# Supplementary material for: Reversible and selective ion intercalation through the top surface of few-layer MoS2
Source: Nat Commun. 2018 Dec 11;9:5289. doi: 10.1038/s41467-018-07710-z (PMC6290021; doi:10.1038/s41467-018-07710-z)
Supplement: Supplementary file 1 — Supplementary Information [file 41467_2018_7710_MOESM1_ESM.pdf]

Supplementary Information

Reversible and selective ion intercalation through the top surface of few-layer MoS_2

Jinsong Zhang[1,2#], Ankun Yang[1#], Xi Wu[3#], Jorik van de Groep[1], Peizhe Tang[4], Shaorui Li[2], Bofei Liu[1], Feifei Shi[1], Jiayu Wan[1], Qitong Li[1], Yongming Sun[1], Zhiyi Lu[1], Xueli Zheng[1], Guangmin Zhou[1], Chun-Lan Wu[1], Shou-Cheng Zhang[4,5], Mark L. Brongersma[1], Jia Li[3], Yi Cui[*1,5]

[1]Department of Materials Science and Engineering, Stanford University, California 94305, USA.

[2]State Key Laboratory of Low Dimensional Quantum Physics, Department of Physics, Tsinghua University, Beijing 100084, P.R. China.

[3]Laboratory for Computational Materials Engineering, Division of Energy and Environment, Graduate School at Shenzhen, Tsinghua University, Shenzhen 518055, P.R. China.

[4]Department of Physics, Stanford University, Stanford, California 94305, USA

[5]Stanford Institute for Materials and Energy Sciences, SLAC National Accelerator Laboratory, 2575 Sand Hill Road, Menlo Park, California 94025, USA.

Correspondence and requests for materials should be addressed to Yi Cui (*email: yicui@stanford.edu)

[#]J.Z., A.Y. and X.W. contributed equally to this work.

Supplementary Figures and Notes

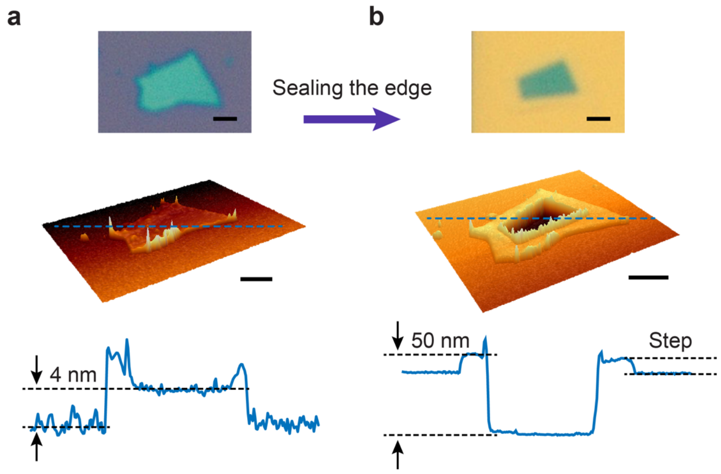

Supplementary Figure 1 The optical and atomic force microscopy images of MoS_2 flake **a** before and **b** after Ti/Au electrode deposition. Scale bars, 2 μm.

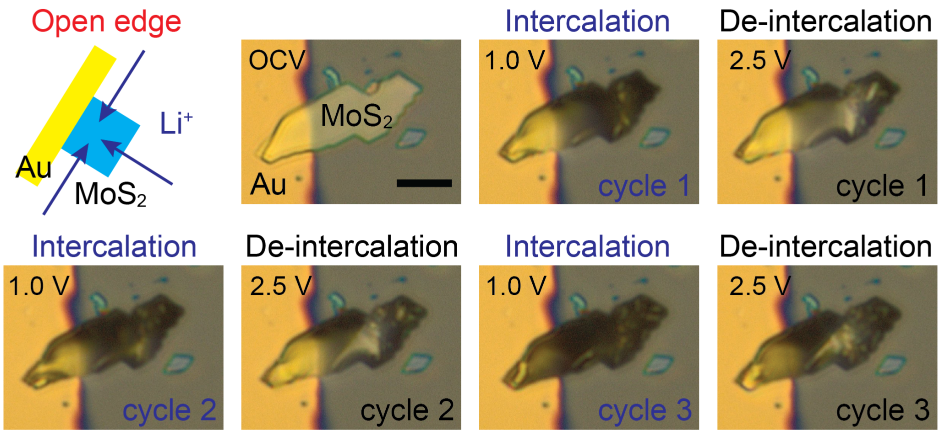

Supplementary Figure 2 Li^+ intercalation and de-intercalation in an open-edge MoS_2. Only a portion of the MoS_2 fake can be recovered and the process was not completely reversible. Scale bar, 5 μm.

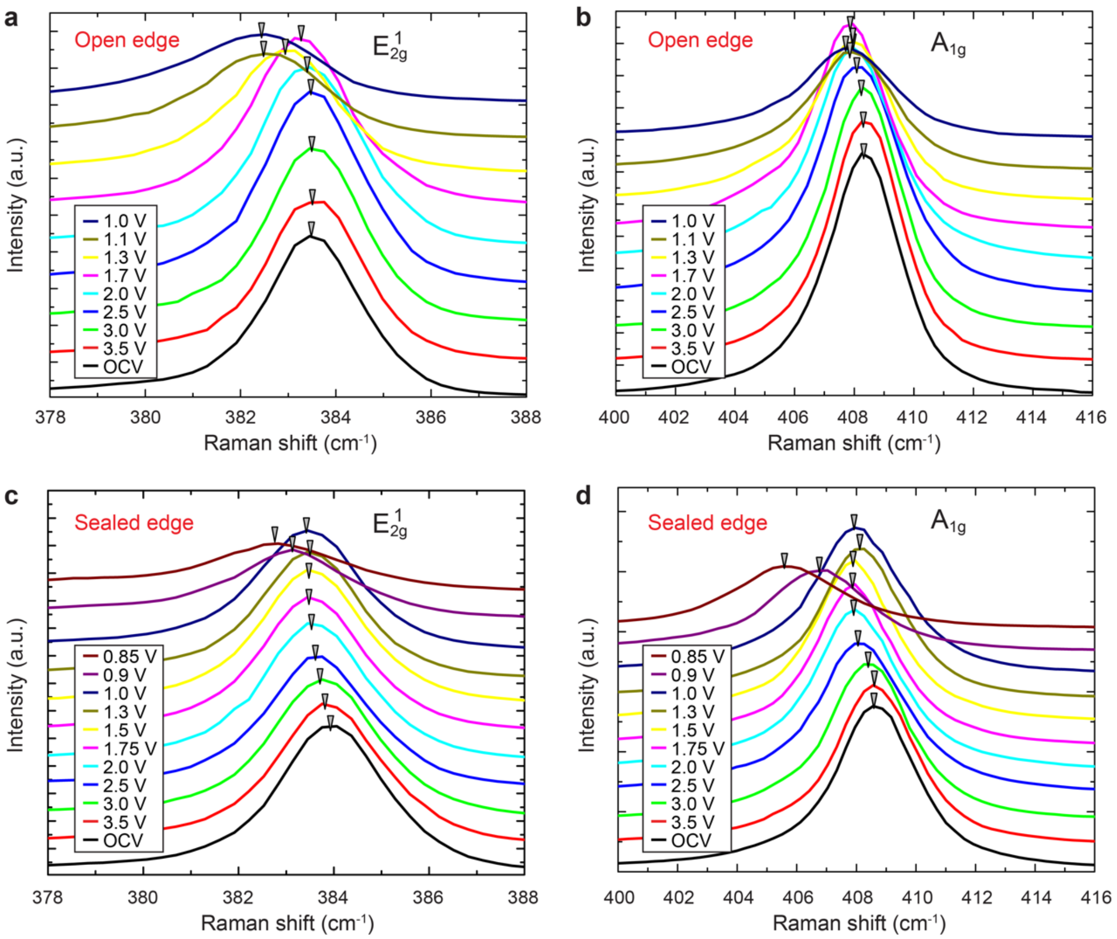

Supplementary Figure 3 Gating of MoS_2 induces slight shift of E_{2g}^{1} and A_{1g} peaks to short wavenumbers for both open edge **a**, **b** and sealed edge **c**, **d** configurations during Li^+ intercalation.

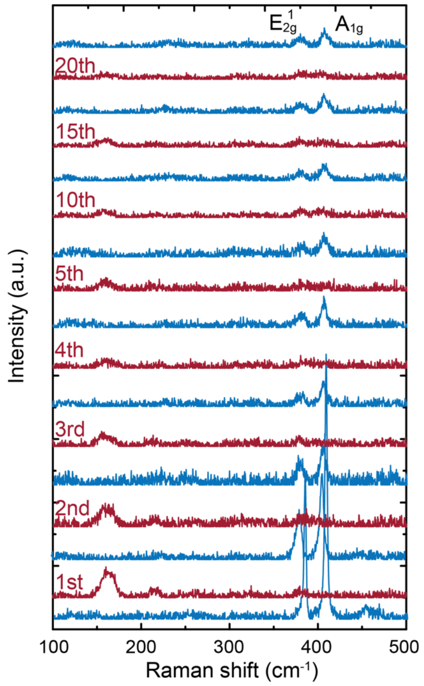

Supplementary Figure 4 Raman spectra of Li^+ intercalation into sealed MoS_2 flakes up to 20 intercalation and de-intercalation cycles. The blue (red) curves represent the Li-deintercalated (intercalated) states.

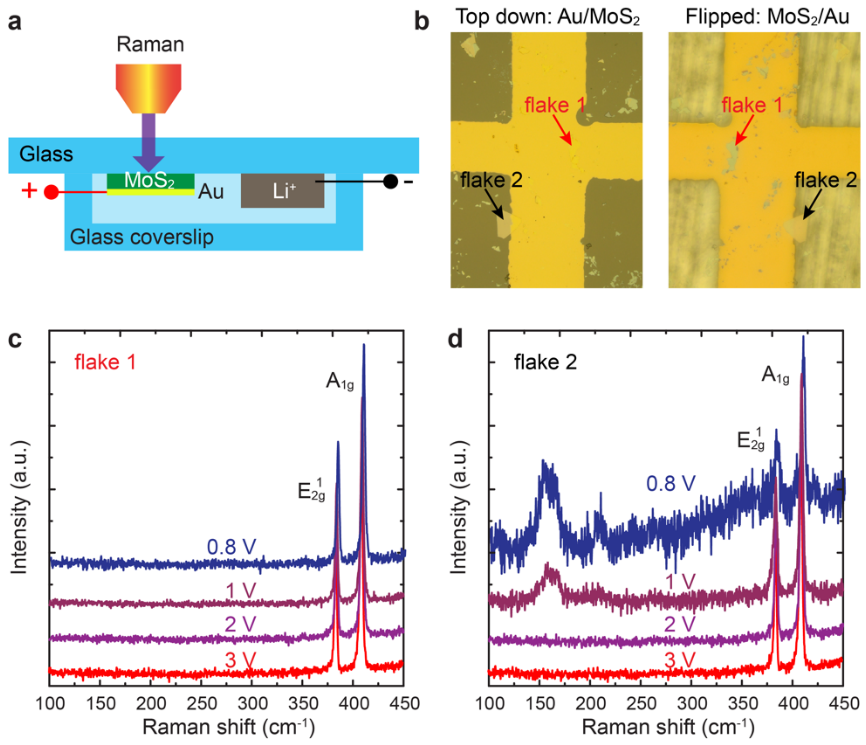

Supplementary Figure 5 The Au electrode can effectively seal the edges of MoS_2. **a** Schematic of the measurement. **b** After flipping, the MoS_2 flakes were on top of Au electrodes which allow in-situ Raman measurements. **c** MoS_2 flake completely covered by Au electrodes was not intercalated until 0.8 V. **d** MoS_2 flake partially covered by Au electrodes was intercalated. The electrodes were Ti/Au (3/50 nm). We note that if the thickness of the flake becomes comparable to the metal thickness, intercalation can still occur due to insufficient coverage.

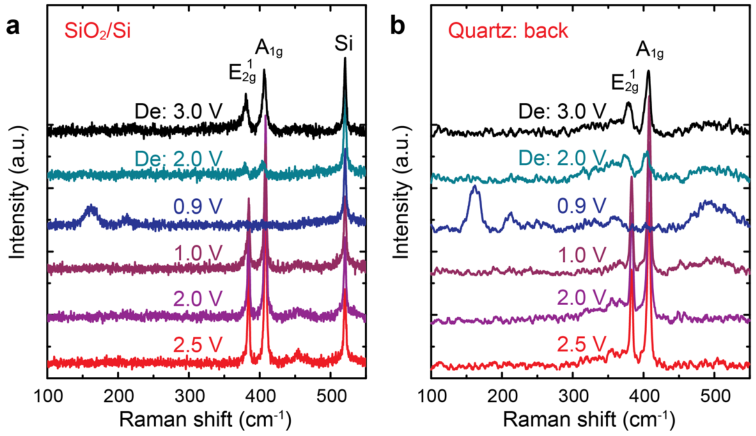

Supplementary Figure 6 Raman spectra of sealed-edge MoS_2 flakes. **a** On SiO_2/Si substrate, the excitation laser light was incident on the top surface of the flake with Si Raman peak as a reference. **b** On quartz substrate, the excitation laser light was incident from the bottom of the device.

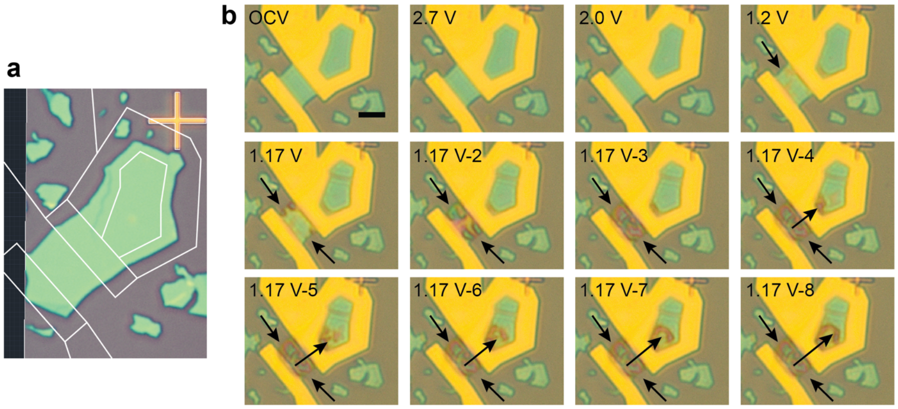

Supplementary Figure 7 a K^+ ion intercalation experiments on a single MoS_2 flake with part of the flake designed as open edge and part of the flake designed as sealed edge. Scale bar, 5 μm. **b** The K^+ ion intercalates through open edge MoS_2 first and then extends into the sealed MoS_2. The arrows indicate the intercalation directions.

DFT calculations

In experiments, we found that alkaline ions can selectively intercalate through the MoS_2 monolayer under moderate voltage, for example, the Li^+ and Na^+ ions can intercalate into the MoS_2 layer reversibly, but K^+ ions cannot. Herein, by using the DFT calculations, we found that the intrinsic defects in MoS_2 play important roles. The alkaline ions can selectively penetrate the MoS_2 layer through these defects.

First, we studied the intercalation process of Li^+, Na^+ and K^+ ions through the defect-free MoS_2 monolayer. The calculated results are summarized in Fig. 4 in the main-text. Due to the mirror symmetry of perfect MoS_2 monolayer, they are equivalent for the most stable adsorption sites on top and bottom sides. Thus, two equivalent peaks can be found in the migration energy path (MEP) for each ion. From our calculations, the Li^+ ion prefers to locate on the top of Mo atom for the top and bottom sides of defect-free MoS_2 monolayer, serving as the initial state (IS) and final state (FS). As shown in Supplementary Figure 9a, there are three steps in the MEP of the Li^+ ion, including the path from Mo-top site (IS) to Mo-hollow site (IS1), the path through Mo-hollow site (IS1 to IS2), and the path from Mo-hollow site (IS2) to Mo-top site (FS). The overall migration barrier is 4.03 eV for the Li^+ ion intercalating through the defect-free MoS_2 monolayer.

For Na^+ and K^+ ions, the difference is negligible between the adsorption energies on Mo-top site and those on Mo-hollow site due to the larger ion radius of Na^+ and K^+, thus the Mo-hollow sites on top and bottom layers of MoS_2 monolayer can be regarded as the most favorable adsorption sites for Na^+ and K^+ ions, serving as IS and FS (Supplementary Figure 9b-c). The migration barriers are 8.32 eV and 13.22 eV for the Na^+ and K^+ ions, respectively. These results show that the Li^+, Na^+ and K^+ ions are difficult to intercalate through the perfect MoS_2 monolayer, even with the additional energy obtained from the applied voltage in experiment.

Secondly, we consider single S vacancy that is the most easily formed point defect in MoS_2 monolayer.[1] The existence of V_S can break the mirror symmetry for both sides of MoS_2 monolayer. On the topside of MoS_2 monolayer, the onsite position of S vacancy is the most favorable adsorption site for Li^+, Na^+ and K^+ ions (regarded as IS), but the Mo-top site is their most favorable adsorption site on the bottom side (regarded as FS). Thus, there are two steps in the MEP of alkaline ions through the MoS_2 monolayer with S vacancy. The first step is for the ions to migrate from S vacancy to Mo-hollow site (IS to IS1). And the second one is from Mo-hollow site to adjacent Mo-top site (IS1 to FS). The corresponding barriers for Li^+, Na^+ and K^+ ions are 4.82, 8.94 and 13.75 eV, respectively (see Supplementary Figure 10).

Another kind of defect V_{S2}, different form V_S, contains two S vacancies and maintains the mirror symmetry of MoS_2 monolayer. The S vacancy is the most favorable adsorption site for Li^+, Na^+ and K^+ ions on both sides of MoS_2 monolayer due to the mirror symmetry, serving as IS and FS. There is only one step in the MEP of alkaline ions through the MoS_2 monolayer. The corresponding barriers are 4.51, 7.53 and 9.14 eV for Li^+, Na^+ and K^+ ions, respectively (see Supplementary Figure 11).

Like V_{S2}, the defect of V_{Mo} also keeps the mirror symmetry, and the Mo vacancy is the most favorable adsorption site for Li^+, Na^+ and K^+ ions on both sides of MoS_2 monolayer. Due to the difference in ion radius for Li^+, Na^+ and K^+, their stable adsorption structures and their MEPs to penetrate through the MoS_2 monolayer with V_{Mo} defect are different. Li^+ ion with smaller radius prefers to be adsorbed at the site of Mo vacancy (see Supplementary Figure 12a). The migration process is a rate-limiting step from Mo vacancy (IS1) to the adjacent Mo-top site (FS), with the barrier of 1.30 eV. Due to the relatively larger radius, Na^+ and K^+ ions prefer to be adsorbed on top of Mo vacancy. Their MEPs to penetrate through Mo vacancy (IS to FS) always contains some

intermediate states. As shown in Supplementary Figure 12b, there is only one intermediate state for Na^+ (IS1, the Na^+ is in the middle of MoS_2 layer), and the migration through the S layer (IS to IS1) is the rate-limiting step, with the barrier of 0.79 eV. However, for K^+ ion, there are two intermediate states, both of which are between the S layer and Mo layer (see Supplementary Figure 12c). The rate-limiting step is the migration of K^+ through the S layer (IS to IS1) with the barrier of 2.46 eV.

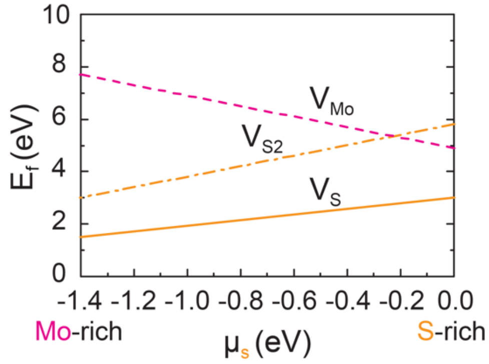

Supplementary Figure 8 Formation energy of S vacancy (V_S), double S vacancy (V_{S2}), Mo vacancy (V_{Mo})

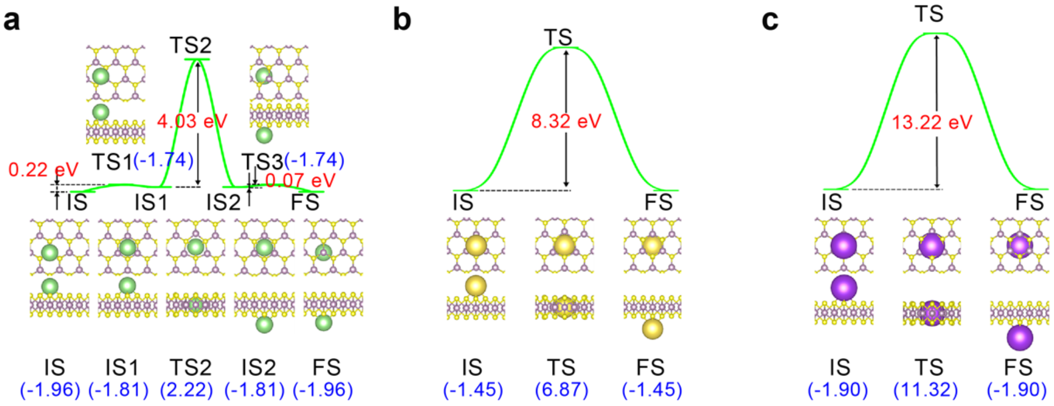

Supplementary Figure 9 The migration of Li^+ (**a**), Na^+ (**b**), and K^+ (**c**) through perfect monolayer MoS_2, respectively. The numbers in blue are the potential in eV for different states. IS: initial state, TS: transition state, FS: final state.

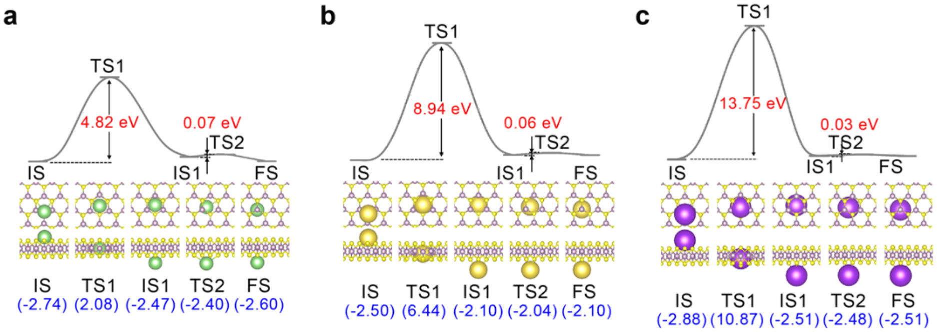

Supplementary Figure 10 The migration of Li^+ (**a**), Na^+ (**b**), and K^+ (**c**) through monolayer MoS_2 with single S vacancy, respectively. The numbers in blue are the potential in eV for different states.

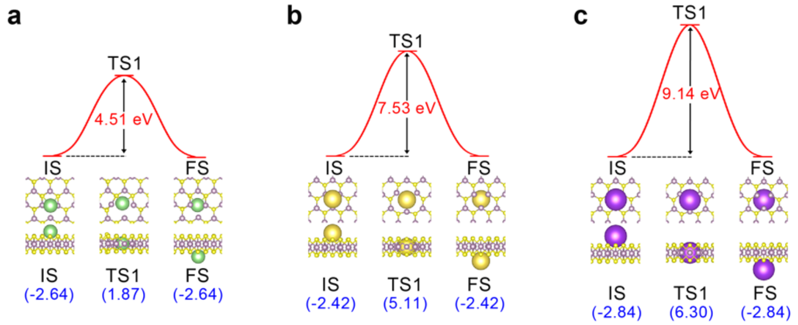

Supplementary Figure 11 The migration of Li^+ (**a**), Na^+ (**b**), and K^+ (**c**) through monolayer MoS_2 with double S vacancy, respectively. The numbers in blue are the potential in eV for different states.

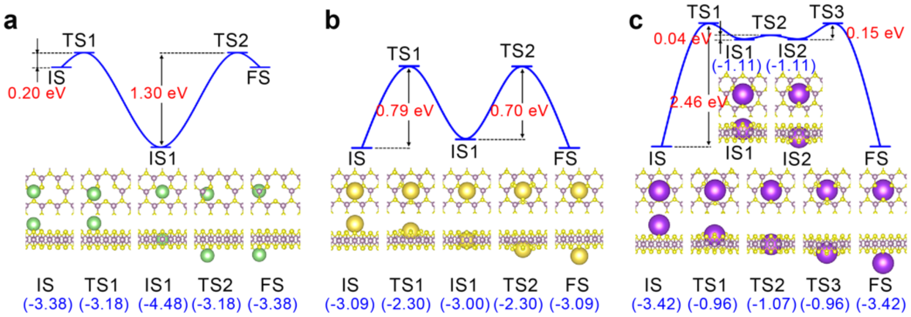

Supplementary Figure 12 a-c The migration of Li^+, Na^+, and K^+ through monolayer MoS_2 with Mo vacancy, respectively. The numbers in blue are the potential in eV for different states.

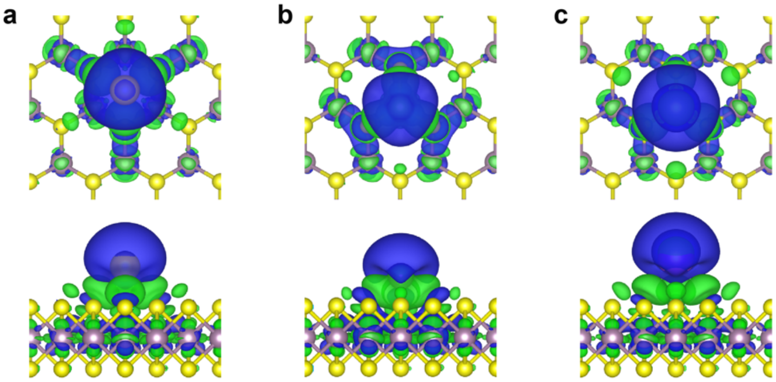

Supplementary Figure 13 Charge distribution when Li, Na, K approach the monolayer MoS_2. When Li, Na, K approach the monolayer MoS_2, charge transfer occurs in the system, green (blue) is the spatial regions gain (loss) in charge. Therefore, during the intercalation process, the ions penetrate through the MoS_2 layer.

Note: In DFT calculation, the intercalation reaction starts from energy favorable adsorption of alkali atom on the MoS_2 surface or vacancy. After the adsorption, the electron transferring from alkali atoms to MoS_2 is around 0.9 e, so these adsorbed alkali atoms can be regarded as alkali ions. Then, we use the migration of adsorbed alkali atom from top of MoS_2 to bottom to simulate the intercalation. Here, we only take monolayer MoS_2 into consideration to investigate the intercalation process of alkali ions. Due to the inversion symmetry of monolayer MoS_2, the de-intercalation process can be regarded as inverse of the migration of adsorbed alkali atom from top of MoS_2 to bottom. In experiment, there is an initial potential to promote the alkali ions to locate on energy favorable sites, then the potential change will force the alkali ions to intercalate through the layer of MoS_2 by appropriate pathways. The electron transfer happens in the process of adsorption of alkali atoms, but there is no conversion between Li ion and Li atom in the process of intercalation and de-intercalation.

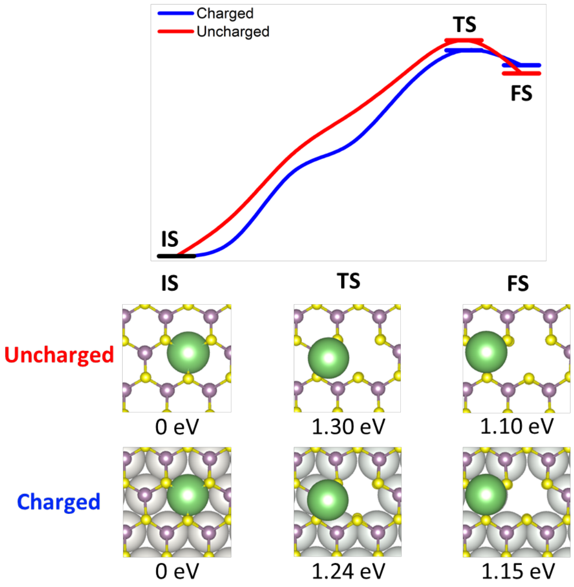

Supplementary Figure 14 Intercalation process of Li^+ on the neutral and charged surface with largest barrier.

Note: The charged surface may play an important role in the migration behavior of alkali ions. To study this, we also tried to simulate a charged surface by placing a layer of full coverage of Li^+ on MoS_2 surface, which could change the work function of the substrate. Without Li^+ coverage, the work function of MoS_2 is 5.63 eV. With additional Li^+ coverage, the work function will change to 4.60 eV. Similar to the additional H^+ coverage to simulate the charged surface for HER/HOR, the Li^+ coverage can also provide a charged surface for the study of Li^+ intercalation through MoS_2. Due to the addition of Li^+ layer, the inversion symmetry of monolayer MoS_2 is broke down. Here, we decided to compare the migration process of intercalation with the largest barrier on a charged surface with that on an uncharged surface. We calculated the migration barrier of Li^+ in the condition of a charged surface. As shown in Supplementary Figure 14, the barrier is 1.24 eV, just 0.06 eV smaller than the neutral surface (barrier as 1.30 eV). We believe the charged surface has limited effects on the intercalation barrier for alkali ions. Therefore, the barrier we obtained in neutral (uncharged) MoS_2 surface can be used to deduce the vital vacancy for selective intercalation of alkali ions.

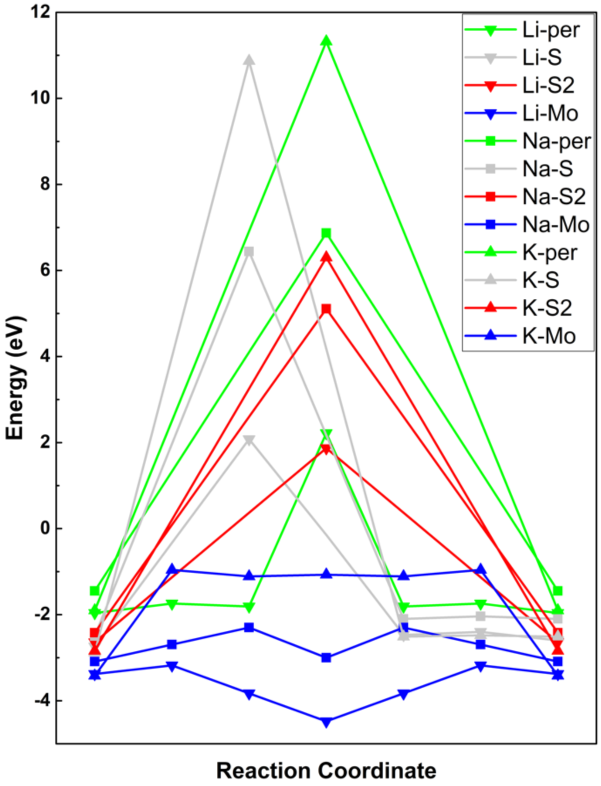

Supplementary Figure 15 Energy profile of the intercalation process of alkali ions through perfect MoS_2, V_S, V_{S2} and V_{Mo}.

Note: When we consider a single layer MoS_2 with and without V_{S2} and V_{Mo}, due to the mirror symmetry with respect to the basal plane, there is no adsorption energy difference between the initial state and final state in the process of intercalation. That is the reason why most of the calculated NEB energy curves are symmetric. For the layered MoS_2 film, there is van der Waals interaction between different layers, so the energy difference between the initial and final states is also very small. In order to show the thermodynamics of electrochemical ion insertion directly, we plot the energy profile of the intercalation process of alkali ions through perfect single-layer MoS_2 or MoS_2 with different defects. As shown in Supplementary Figure 15, only V_S (in gray line) breaks the mirror symmetry of the MoS_2 surface, but the energy difference between initial state and final state is negligible compared to its migration barrier. Therefore, the kinetic effect plays vital role in the process of intercalation rather than the thermodynamic effect.

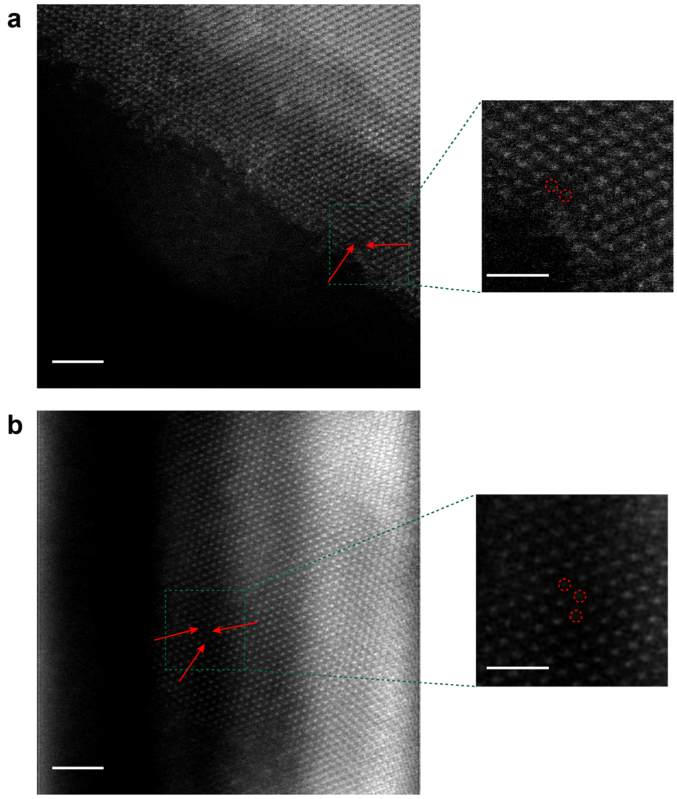

Supplementary Figure 16 HAADF-STEM images of exfoliated MoS_2, the red dashed circles indicate Mo vacancies. Scale bars, 2 nm. Scale bars in enlarged images, 1 nm.

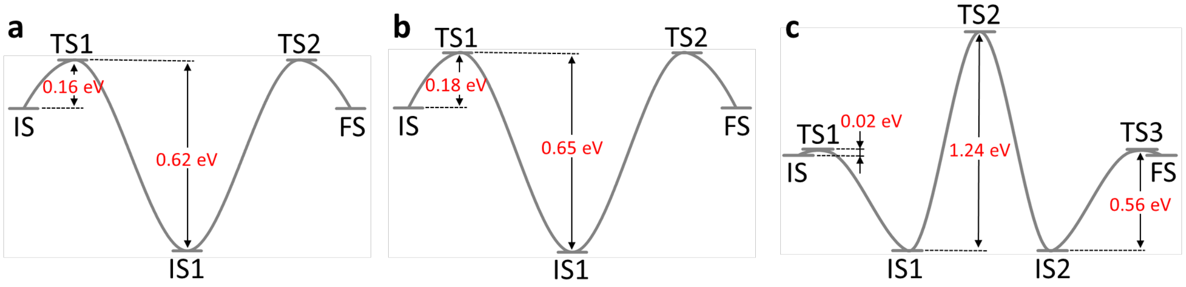

Supplementary Figure 17 The migration of (**a**) Li^{+}, (**b**) Na^{+} and (**c**) K^{+} through V_{MoS6}.

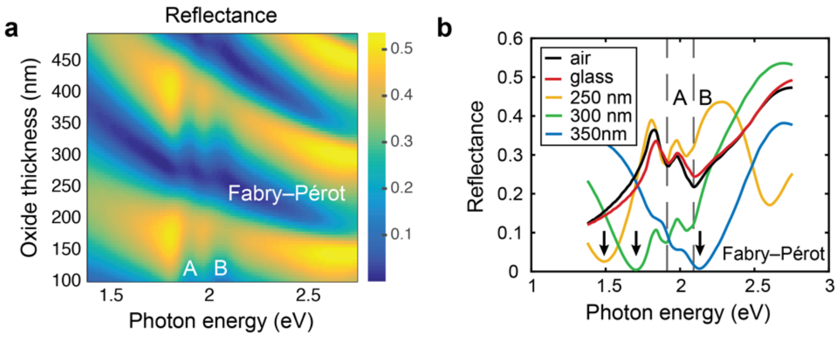

Supplementary Figure 18 Interplay of Fabry–Pérot resonance with excitons A and B. **a** Fabry–Pérot resonance and excitons A and B as a function of oxide thickness. Due to overlap in energy, Fabry–Pérot resonance (dispersive as a function of oxide thickness) and excitonic transitions A and B (intrinsic properties of the MoS_2, independent of the oxide thickness) show interactions with each other. The color scale of reflectance is shown on the right. **b** Fabry–Pérot resonance sweeps across excitonic transitions A and B showing typical Fano-like lineshape. We used 300-nm SiO_2/Si substrate, where exciton A is likely buried in the broad Fabry–Pérot resonance. In contrast, the MoS_2 flake in air or on glass show clearly only excitonic transitions A and B without Fabry–Pérot resonance. We approximated some parameters in the simulations which may cause the slight difference from the experimental results. Specifically, the refractive index of the liquid electrolyte and SiO_2 were approximated as 1.4 and 1.46, respectively; the thickness of the MoS_2 flake was 5 nm; and the optical constants of monolayer MoS_2 were used.[2]

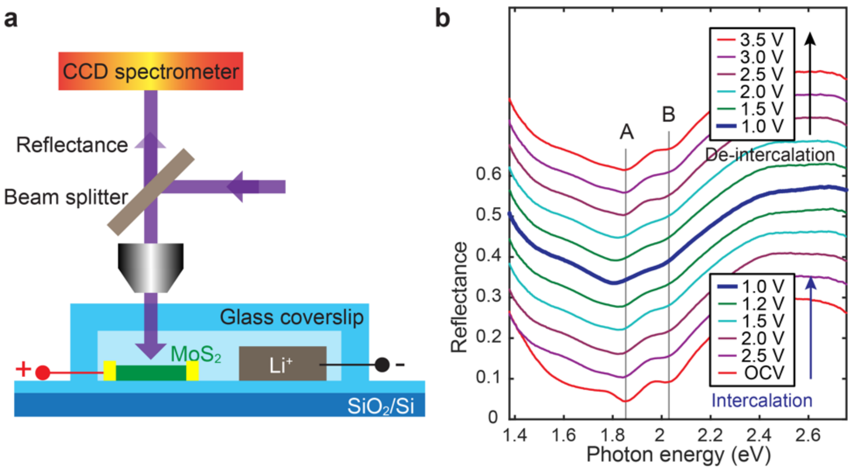

Supplementary Figure 19 Reversible control of optical properties of MoS_2 via Li^+ intercalation. **a** Schematic of the optical measurement. **b** Gradual reflectance change of MoS_2 on SiO_2/Si via Li^+ intercalation (spectra offset for visibility). From bottom red line to middle blue line: ion intercalation; from middle blue line to top red line: ion de-intercalation. For the pristine flake, two reflectance dips were observed at around 1.85 eV and 2.02 eV, also known as A and B excitons. The slight differences between the exciton energies in Supplementary Figure 18 and Fig. 5 in the main text were due to the difference in the dielectric environment.[3] Upon intercalation, A exciton showed a minor redshift (possibly also damping in intensity) and B exciton exhibited a clear damping in intensity which might be the overall effect of bandgap shrinking due to the structural change (red-shift) and the decrease in binding energy resulted from the doped electrons during Li^+ intercalation.

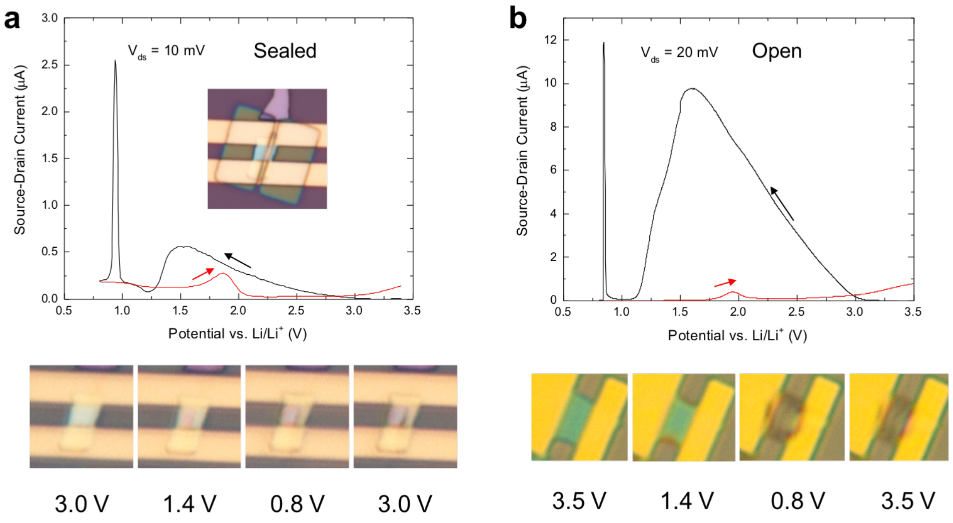

Supplementary Figure 20 Transport property comparison of Li^+ intercalation in sealed and open-edge MoS_2. **a** Source-drain current scan of Li^+ intercalation into MoS_2 sealed with SiO_2. **b** Source-drain current scan of Li^+ intercalation into open-edge MoS_2.

Note: To study the transport properties of the top-surface intercalated devices, we need to seal the MoS_2 flakes using dielectric materials instead of Au. We have fabricated the devices with insulating SiO_2 (100 nm thick) to seal the edges of MoS_2 as shown in the inset of Supplementary Figure 20a. Compared with the transport data of open-edge MoS_2 (Supplementary Figure 20b, the same device as in Fig. 2b in the main text), the drain-source current both showed similar dependence on the potentials vs Li/Li^+. When the potential was first scanned from 3.5 V to 1.5 V, the increase of current results from Li^+ gating effect. From 1.5 V to 1.0 V, Li^+ started to intercalate, and current dropped possibly due to the creation of more defects from Li^+ intercalation. Between 1.0 V and 0.8 V, the current showed a sharp peak, which is probably due to the phase transition from semiconducting 2H phase to metallic 1T phase, giving rise to the drastic current increase. The degradation of Li^+-intercalated MoS_2, such as exfoliation or other irreversible reactions, may result in the prominent decrease of current.

Due to the similarity of the current curves between sealed- and open-edge devices, we can conclude that the SiO_2 sealant didn't play an essential role during Li^+ intercalation. This is because the SiO_2 layer may be cracked or peeled off during the Li^+ intercalation due to the c-axis expansion and low malleability of SiO_2 compared with Au metal. From the captured images (lower panel of Supplementary Figure 20a), we found that the original morphology of the SiO_2-sealed MoS_2 device cannot be restored after the retraction of voltage to 3.0 V, which further demonstrated the instability of SiO_2 sealant during Li^+ intercalation. We also tried to use LiF to seal MoS_2, but cannot obtain any positive results at this stage. Thus, we realized that it is extremely difficult to find an insulating material with high malleability, electrochemical stability and high affinity to seal MoS_2 edges at this point.

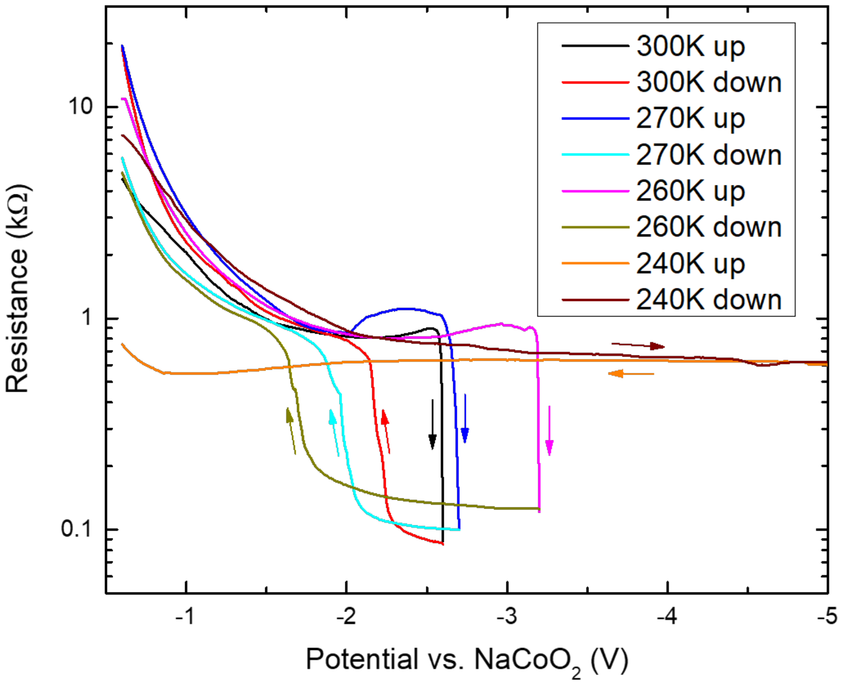

Supplementary Figure 21 The four-probe resistance measurements at different temperatures with respect to the potentials *vs.* $NaCoO_2$. The arrows indicate the scanning directions.

Note: To further check the functionality of Na^+ electrolyte at low temperatures, we performed the 4-porbe resistance measurements at different temperatures. As can be seen in Supplementary Figure 18, the resistance of the MoS_2 flake showed a dramatic decrease when the Na^+ was intercalated, and then recovered when Na^+ was de-intercalated. However, higher (more negative) intercalation potentials were needed at lower temperatures, that is -2.7 V at 270 K and -3.2 V at 260 K. At 240 K, the Na^+ cannot be intercalated even up to -5 V. Therefore, we had to apply the potential at room temperature to perform the intercalation, and then lowered the temperature and kept the potentials to study the carrier density changes.

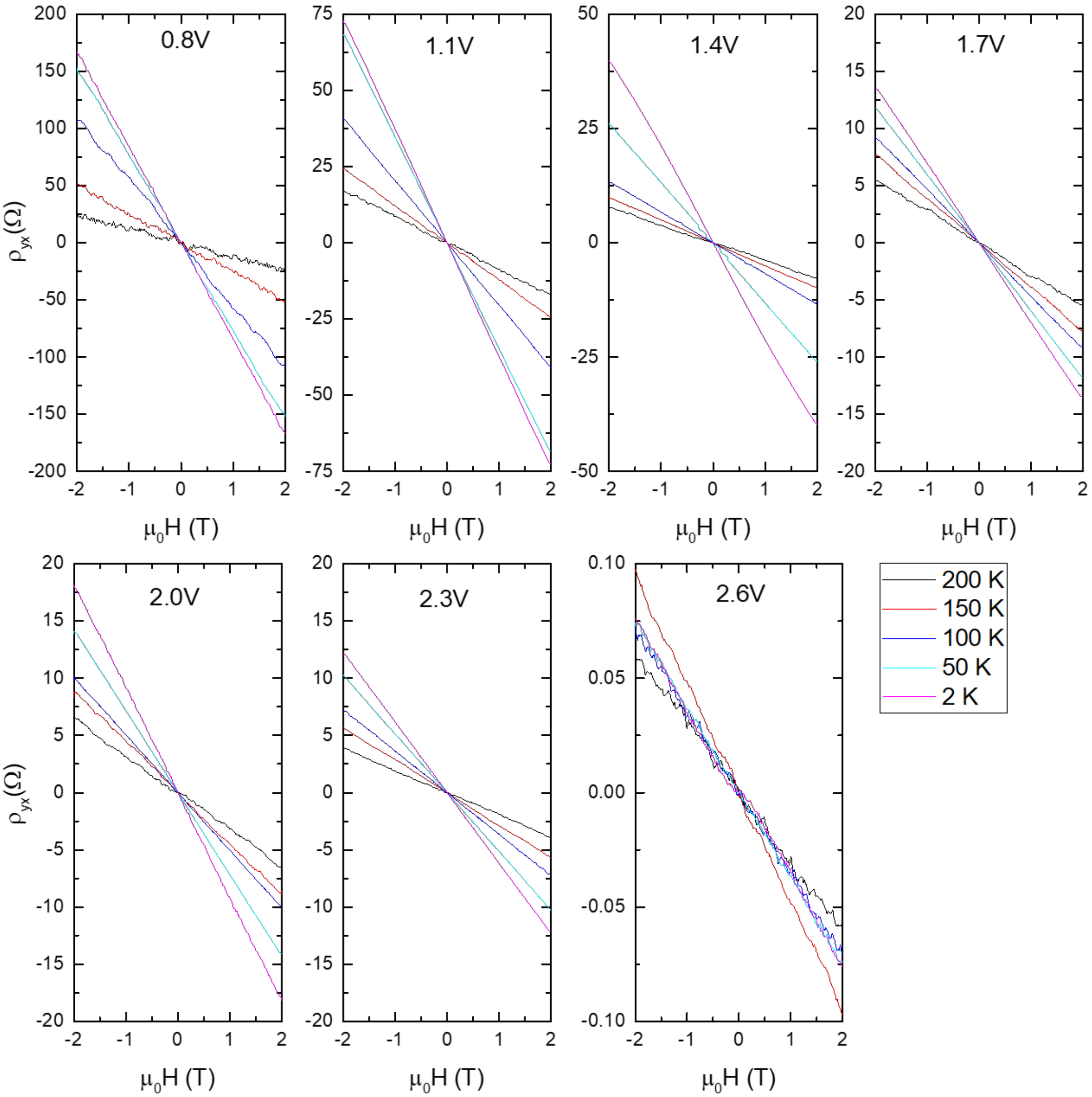

Supplementary Figure 22 The Hall resistance as a function of the magnetic field at different temperatures and potentials *vs.* $NaCoO_2$ counter electrode. We only recorded the Hall signals below 200 K because it took too much time to stabilize at temperatures above freezing point.

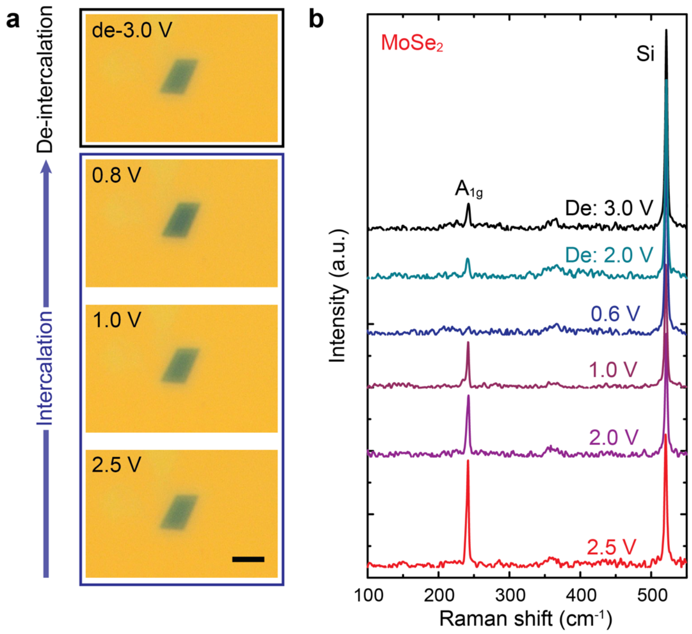

Supplementary Figure 23 Intercalation of sealed-edge $MoSe_2$. **a** In-situ optical microscopy images of Li intercalation into $MoSe_2$ through top surface. Scale bar, 5 μm. **b** In-situ Raman spectra of Li intercalation into $MoSe_2$ with sealed edge.

References

1. Zhou, W. *et al.* Intrinsic Structural Defects in Monolayer Molybdenum Disulfide. *Nano Letters* **13,** 2615–2622 (2013).
2. Liu, H.-L. *et al.* Optical properties of monolayer transition metal dichalcogenides probed by spectroscopic ellipsometry. *Appl. Phys. Lett.* **105,** 201905 (2014).
3. Lin, Y. *et al.* Dielectric Screening of Excitons and Trions in Single-Layer MoS 2. *Nano Letters* **14,** 5569–5576 (2014).
